# A model analysis of static stress in the vestibular membranes

**DOI:** 10.1186/1742-4682-6-19

**Published:** 2009-09-01

**Authors:** Daniel J Pender

**Affiliations:** 1 Department of Otolaryngology, Columbia University Medical Center New York, USA

## Abstract

**Background:**

The scheme of the core vestibular membranes, consisting of serially connected utricle, ampulla and semicircular canal, first appeared hundreds of millions of years ago in primitive fish and has remained largely unchanged during the subsequent course of evolution. The labyrinths of higher organisms build on this core structure, with the addition of the phylogenetically newer membrane structures, namely, saccule, lagena and cochlea. An analysis of static stress in these core vestibular membranes may contribute to a better understanding of the role of stress in the evolution of derivative membrane structures over the long term as well as the short-term membrane distortions seen in Meniere's disease.

**Methods:**

A model of these core vestibular membranes is proposed in order to analyze the distribution of stress in the walls of the component chambers. The model uses basic geometrical elements of hollow cylinders and spheres to emulate the actual structures. These model elements lend themselves to a mathematical analysis of static stress in their membranes.

**Results:**

Hoop stress, akin to the stress in hoops used to reinforce barrel walls, is found to be the predominant stress in the model membranes. The level of hoop stress depends not only on pressure but as well on a geometric stress factor that incorporates membrane shape, thickness and curvature. This result implies that hoop stress may be unevenly distributed in the membranes of the several vestibular chambers due to variations in these dimensional parameters. These results provide a theoretical framework for appraising hoop stress levels in any vestibular labyrinth whose dimensions are known.

**Conclusion:**

Static hoop stress disparities are likely to exist in the vestibular membranes given their complex physical configurations. Such stress disparities may contribute to the development of membrane pathologies as seen in Meniere's Disease. They may also factor in the evolutionary development of other derivative membrane structures such as the saccule, the lagena, and the cochlea found in higher animals.

## Background

**Figure 1:**
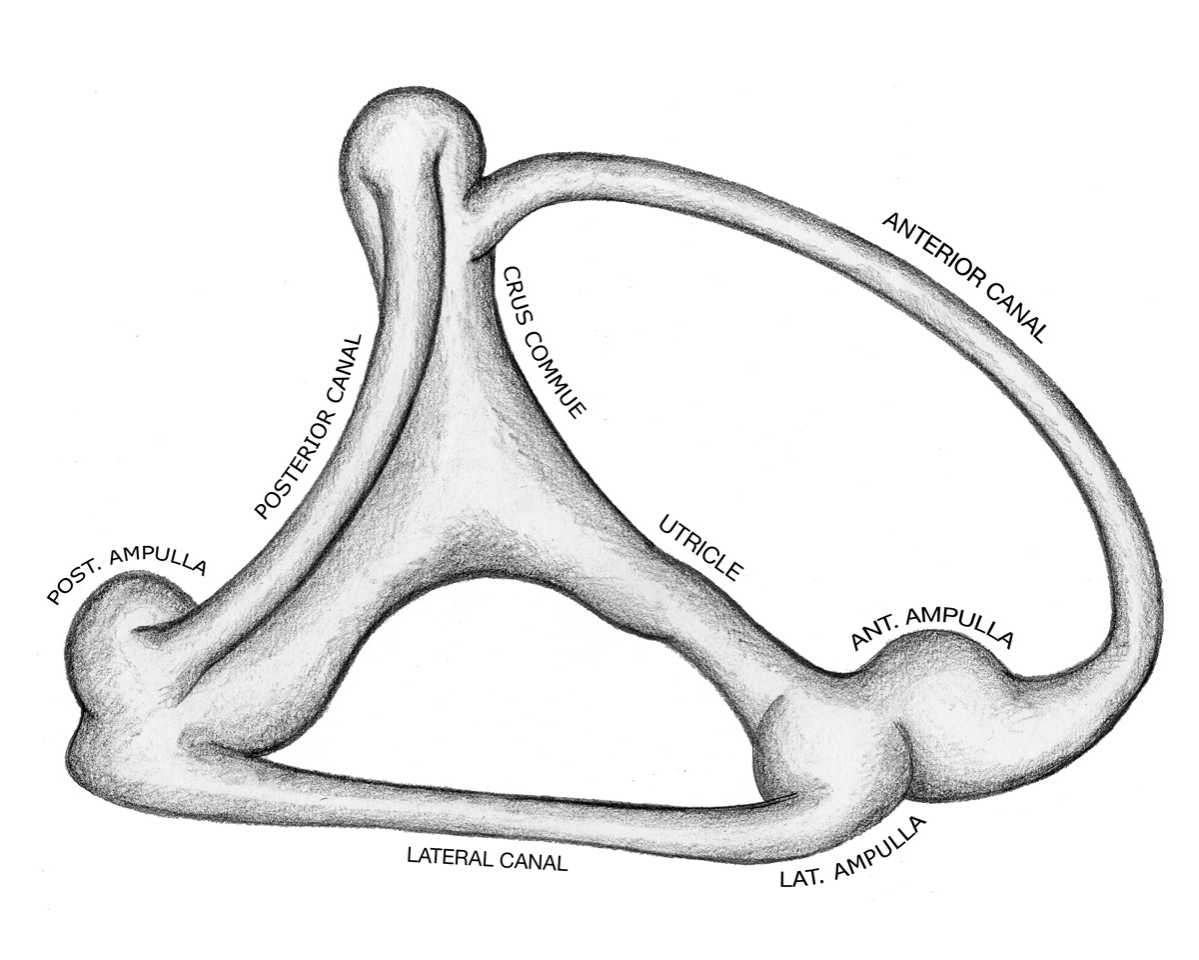
**Vestibular Labyrinth in the toadfish (after Ghanem)**. The toadfish vestibular labyrinth has three semicircular canals and three ampullas connected to a common utricle.

The core vestibular membranes in vertebrate and proto-vertebrate fish consist of one or more ducts connected with a number of chambers that house sensory epithelia concerned with detection of acceleration [1] as depicted in Figure 1. These core membrane structures are the semicircular canal, the ampulla, and the utricle. This arrangement first appears in primitive fish during the Ordovician period some five hundred million years ago [2]. Its scheme of interconnected canals and chambers is common to all subsequent animal labyrinths. This feature suggests that a model analysis might be helpful in understanding the stability of these core membranes and their potential for dysfunction under stress. Areas subject to stress concentration could explain why membranes evolve appendages, such as lagena, saccule, and cochlea, or fail outright, as in Meniere's Disease.

To this end, a theoretical model is proposed that emulates the vestibular chambers with basic geometric shapes that are mathematically tractable. Such shapes lend themselves readily to a static force analysis of the relationship between trans-mural pressure and the reactive stresses induced in the various membrane walls [3]. The model will be limited to the utriculo-ampullo- canal complex from which the more elaborate structural configurations found in higher forms derive.

## Methods

In order to create a model of the basic vestibular labyrinth that will provide insight into the biomechanics of the component membranes, it is necessary to:

(a) evaluate the configuration of the physical membranes to be emulated

(b) select ideal shapes for the model membranes

(c) describe the general mechanics of membranes

(d) define the determinants of membrane stress

### The Physical Membranes

Stress is known to be dependent on membrane thickness and membrane curvature [3]. Evaluation of these physical parameters is thus critical to the model emulation.

Thickness of the vestibular membranes is due mainly to a dominant layer of collagen sandwiched between thin sheets of epithelial and mesothelial cells that form the inner and outer surfaces of the membrane [4]. This collagen layer appears to be of relatively even thickness histologically [1,4]. This prominent collagen layer is assumed to bear the brunt of the stress load in the vestibular membranes. However in the absence of this collagen layer the very thin basement membrane acting as a support for epithelial cells facing the endolymphatic compartment would become the load bearing structure [5].

**Figure 2:**
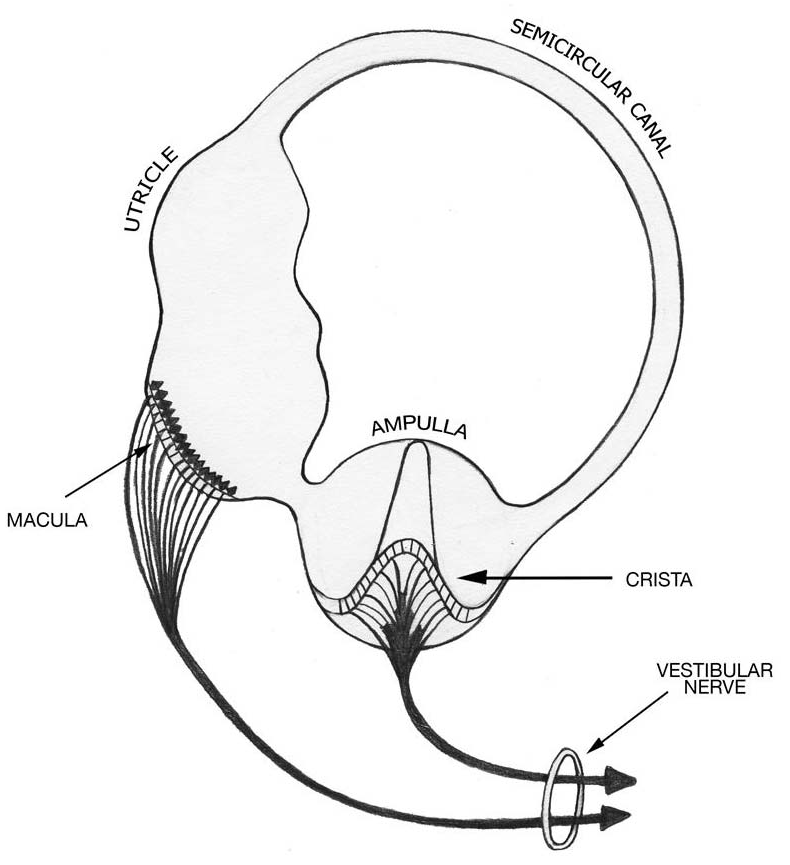
**Scheme of the Vestibular Labyrinth (after Melville-Jones)**. This schematic highlights the serial organization of the three component chambers in the basic vestibular labyrinth.

The shape of the membranes in the three vestibular structures can be estimated from their descriptions and depictions in the literature. The anatomical term semicircular canal speaks to its toroidal shape. Ampulla is the diminutive of the Latin 'amphora', a quasi-spherical vessel with handles used for wine or perfume. Utriculus is the diminutive of uterus from the Sanskrit 'udarum' meaning belly. It refers to pouch-like enlargement of the oviduct for containing and nourishing the fertilized egg. Its shape in the ear has been described as a tubular [4]. The full array of animal labyrinths preserved and depicted in stereo-photographs by AA Gray permit ready visual confirmation of these various vestibular chamber shapes [6]. The anatomical drawings of Max Brodel depict these structures in a congruent manner [7]. These chamber morphologies are generally consistent with a schematic drawing of the utriculo-ampullo- canal complex that highlights their relative sizes and shapes and their serial interconnection [8], as seen in Figure 2. Finally, digital reconstructions of the toadfish vestibular labyrinth [1] confirm the toroidal, tubular and spherical shapes of the semicircular canal, the utricle and the ampulla, respectively.

### The Model Membranes

The histological features noted above suggest that as a first approximation the membranes can be modeled as relatively uniform and isotropic. (A more complex model would be needed to account for the fibrous nature of the collagen layer along with its implied anisotropy. This is beyond the scope of the current study.)

The anatomical descriptions noted above suggest the spherical and cylindrical shapes for the model chambers. Therefore in the current model, the narrow toroidal shape of the semicircular canal will be construed to be a long thin straight cylinder, the dome of the ampulla a sphere, and the utricle a cylindrical tube. Such axially symmetric shapes permit ready determination of membrane curvature, since curvature is defined as the inverse of radius. This feature lends itself to ready data acquisition from actual tissues when measurements are needed for numerical calculations.

### General Membrane Mechanics

**Figure 3:**
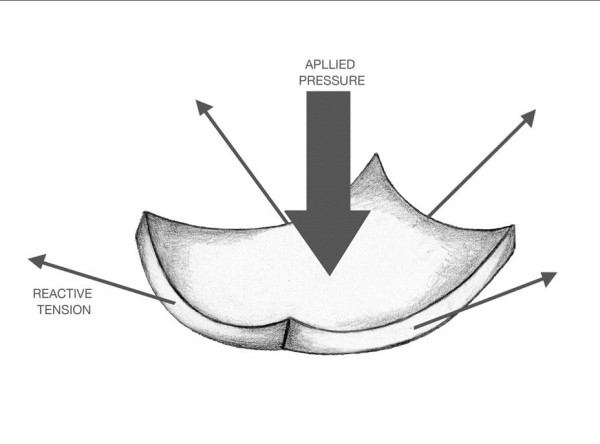
**Forces acting on a spherical membrane element**. Trans-mural pressure induces a reactive tension in the plane of the membrane.

In simple compression of a membrane, a reactive stress is induced equal and opposite to the pressure applied. In such a situation the reactive stress is co-linear with that applied and unmagnified, i.e. the ratio of reactive stress to applied pressure would be unity. However, in a suspended thin membrane, e.g. a trampoline, the trans-mural pressure is resisted by two orthogonal tensile stresses that develop in the curved plane of the membrane as it deforms as seen in Figure 3. The relative magnitude of these stresses in the proposed model can be estimated using the constitutive membrane equation. This equation can be applied to any thin wall biological structure that offers no bending resistance [3]. This requirement implies that the membrane stresses are purely tensile or compressive and thus relatively uniform.

The membrane equation is derived in basic mechanics through a balance of forces acting on a membrane element [9]. For any given point in a membrane with dual curvature where pressure is balanced by two intramural stresses, the membrane equation becomes:

(1)

Where **'p' **is the trans-mural pressure level,

Where **'t_x_' **and **'t_y_' **are the intramural stresses in the two coordinate directions,

Where **'w' **is the membrane wall thickness at the point in question, and

Where **'r_x_' **and **'r_y_' **are the two orthogonal radii of curvature of the membrane segment.

This equation shows that the two stress components are modulated by the dimensions of radius and wall thickness. The relative magnitude of these stress component terms can vary from point to point in the membrane as long as the sum remains constant and equal to the trans-mural pressure. It should be noted that stress 't' can also be negative, ie compressive, and that curvature ('1/r') can also be negative, i.e. reversed.

**Figure 4:**
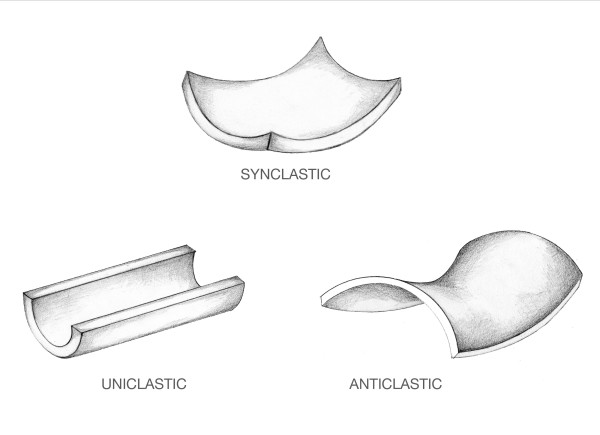
**Membrane Configurations**. There are three possible shapes that a membrane may assume.

Positive and negative membrane curvatures are shown in Figure 4. When both curvatures of a differential element bend in the same direction, as in a trampoline, the membrane is designated synclastic, and its tensile forces are additive in opposing trans-mural pressure. Synclastic membrane elements are found in the spherical ampulla. When curvatures are of opposite sign, as in a saddle, the membrane configuration is designated anticlastic with its tensile forces oppositional and pressure balanced by their net difference. Anticlastic membrane areas are found on the inner surface of the semicircular canal. When there is only one curvature, the configuration may be described as uniclastic. Such areas are encountered in the cylindrical utricle.

### Membrane Stress Determinants

The sphere and cylinder model shapes proposed above are part of the ellipsoid family, a general figure that lends itself to mathematical analysis.

**Figure 5:**
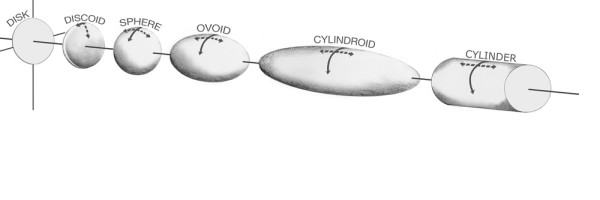
**Family of Ellipsoids with circular cross-section**. As a cylindrical shape is approached, the gradual lessening of axial curvature necessitates an increasing hoop stress.

Ellipsoids with constant circular cross section at the equator of radius **'r' **and increasing axial length **'b' **constitute a continuum that ranges from a round disk to an infinite cylinder shown in Figure 5. The general expression for such a family of ellipsoids is given in Equation 2.

(2)

As the axial dimension grows, the ellipsoid progresses from disk through discoid to sphere and then from ovoid through cylindroid and asymptotically to cylinder.

Several features should be noted about stresses in ellipsoids as illustrated in Figure 5. Membrane stress aligned with the equator can be referred to as 'hoop' stress, akin to the stress in hoops used to reinforce barrel walls. Membrane stress running from pole to pole can be designated as 'meridional' akin to the prime meridian line of longitude. In flattened discoid shapes (oblate spheroids), meridional stress is always the dominant stress and will always be maximal at the poles; the flatter the discoid, the higher the polar meridional stress, since flat membranes cannot offer resistance to trans-mural pressure. None of the vestibular chambers under consideration appears to be of this configuration. In the perfect spherical shape, the geometrical symmetry implies that hoop stress and meridional stress must be equal. Beyond this, in elongated ellipsoid shapes (prolate spheroids), i.e. in the model range from sphere to cylinder, hoop stress becomes the dominant stress and is always maximum at the equator where girth is greatest and hoop curvature least. As the sphere lengthens into an ovoid shape and then a cylindroid, the axial curvature lessens and with it the contribution of meridional stress to resisting trans-mural pressure. As a consequence, in ellipsoid figures of constant diameter at the equator, the membrane equation implies that hoop stress at the equator gradually increases with axial length until it reaches the maximum associated with a uniclastic cylindrical shape [9].

Mathematically, these considerations are implicit in Equation 3 that describes the hoop stress at the equator of a family of ellipsoids of constant equatorial diameter [9] in terms of its several parameters:

(3)

Where 't_hoop_' designates hoop stress at the equator

'r' designates hoop radius at the equator

'w' designates membrane thickness

'b' designates the axial semimajor dimension of the the ellipsoid.

'p' designates transmural pressure.

Note that when 'r' equals 'b' the figure is a sphere of radius 'r' and Equation 3 becomes

(4)

When 'b' is infinite the figure becomes a cylinder of radius 'r' and Equation 3 becomes

(5)

It should be noted that other smaller stresses can exist in the model membranes in addition to predominant hoop stresses. Maximum membrane shear stress exists in a 45 degrees plane and is one half the difference between the two principal stresses acting on a membrane element [9].

Radial stress, oriented perpendicular to the membrane surface exists only in thicker membranes [10] but is negligible in thin membranes with no bending resistance [3]. Thinness of a membrane is reflected in the ratio of radius to wall thickness. Values greater than five indicate that the diameter of the chamber is ten fold that of the wall thickness and constitute an engineering criteria for 'thin' for purposes of this analysis [10]. Thick membranes with a value less than five are more apt to offer bending resistance and are associated with increasingly significant levels of radial stress as predicted theoretically by the Lame equations [3], and their stress analyses would depart from that presented here.

Thus, in the model, hoop stress is the only stress in the spherical chambers while in the cylindrical chambers it is the main stress accompanied by an axial component half its magnitude and a shear stress component one quarter its magnitude. Therefore the subsequent analysis will focus on hoop stresses as being the dominant tensile stress in all model chambers with the understanding that smaller axial and shear stresses are also present in the cylindrical model elements.

## Results

**Figure 6:**
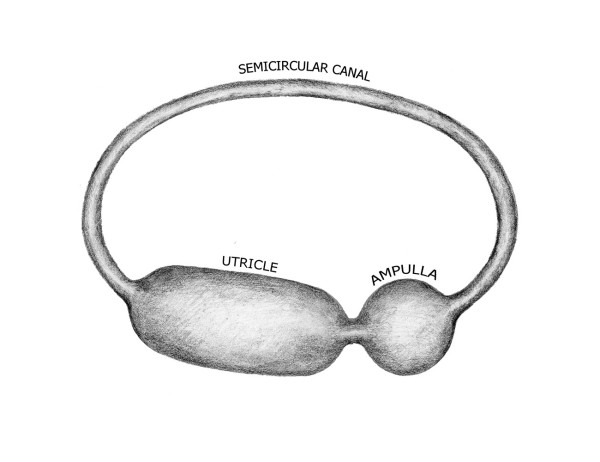
**Model of the Vestibular Membranes**. The building blocks employed are cylinders and spheres.

Taken together the configurations of the several vestibular chambers suggest a basic model of the vestibular membranes that is depicted in Figure 6. It is in essence a conjoined utriculo-ampullo-canal complex, using simple shapes as building blocks to emulate the component structures. The model as shown reflects the fact that the semicircular canal always has the least diameter but defers on the relative sizes of utricle and ampulla. The model entails no dimensions and is intended to depict only the interconnection of the component chambers and their basic geometric shapes. All are in open communication internally allowing a constant transmural pressure throughout. Given these considerations, this basic model is constructed so as to approximate the scheme of the vestibular membranes. This model can be used to investigate hoop stress in the vestibular membranes of any species to the extent that the model is built of sufficient components to reflect the species' anatomy and actual dimensions are known. As shown, it most closely reflects the membranous labyrinth of the primitive hagfish that has only one semicircular canal. In order to model the river lamprey, a second canal and ampulla would need be added to the utricle and to model the oyster toadfish, a third canal and ampulla.

As to the determinants of hoop stress levels in the model membranes, inspection of Equation 3 indicates that it is of the form:

(6)

Where (s) represents membrane shape, e.g. (1 - r^2^/2b^2^) for an ellipsoid.

Where (r) represents membrane radius of curvature (chamber size)

Where (w) represents membrane thickness

Where (p) represents transmural pressure.

Since both **'s' **and **'r/w' **are dimensional in nature, they may be conveniently combined and viewed as a composite geometric stress factor that controls the degree of hoop stress induced by the transmural pressure (p). Thus for equatorial hoop stress in an ellipsoid the geometrical stress factor would be **(1 - r^2^/2b^2^) (r/w)**.

Thus, in general, equatorial hoop stress in the model membranes can be expressed as a function of pressure, modified by a geometric stress factor **'(s) (r/w)' **composed of membrane shape **'s'**, membrane chamber size **'r'**, membrane wall thickness **'w'**, as shown in Equation 6. It should be noted that any comparison of hoop stress between chambers, as occurs when stresses are normalized, reduces to a simple ratio of geometric stress factors since pressure is constant throughout the chambers.

## Discussion

Models have long been used to gain insight into the behavior of complex and unwieldy physical systems [11]. Dynamic vestibular models concerned with endolymphatic flow and its transductance by the sensory epithelia have been reported [12,13]. The current study presents a model of static hoop stress in the investing vestibular membranes. These membranes constitute an irregularly shaped complex of interconnected chambers (utricle, ampulla and semicircular canal) [1]. Modeling of these chambers individually with basic geometric shapes and joining them in series permits an emulation of the core vestibular membranes.

This model can be analyzed to investigate disparities in membrane stress in the vestibular chambers. This analysis shows that static hoop stress is linearly dependent on trans-mural pressure, as well as a dimensional factor indicative of membrane geometry. Chamber shape, size and wall thickness all modulate the membrane stress reaction through the agency of a geometric stress factor according to the Equation 6. This geometric stress factor **'(s)(r/w)' **magnifies or reduces the effect of pressure on reactive hoop stress in the membrane walls. This implies that while pressure may be equal throughout the model labyrinth, hoop stress will vary from point to point according to the local membrane geometry. Evaluation of these several stress determinants is thus critical to understanding stress disparities that can provoke membrane deformation and thus potentially play a role in evolutionary and disease processes.

**Pressure **is the active determinant of membrane hoop stress and reflects ongoing physiological processes in the endolymphatic tissues. In a closed endolymphatic system this pressure can be expected to depend on the net balance between fluid secretion and resorption. Processes that interfere with fluid secretion such as gentamicin induced dark cell toxicity [14] can be expected to reduce pressure while processes that interfere with resorption such as vestibular aqueduct ablation [15] can be expected to raise pressure. This analysis shows that whatever the equilibrium pressure might be, hoop stress in any particular chamber will be directly proportional to that pressure.

The **geometric stress factor**, as noted above, is a composite of three passive determinants of hoop stress that act in concert to modulate the effect of pressure. A qualitative overview of this stress modulating effect can be appreciated by considering permutations of these three determinants. When values are arbitrarily limited to cylinder or sphere for shape, large or small for size, and thick or thin for membrane thickness, then eight overall permutations exist. Among these a small thick walled sphere would result in a minimal geometric stress factor while a large thin walled cylinder would result in a maximal one. Within this range intermediate values would be encountered reflecting the variations in membrane shape size and thickness. To get a more quantitative estimate of the effect of the geometric stress factor on membrane hoop stress the individual contributions of the three components must be adduced.

**Membrane shape **is a passive determinant of hoop stress in that its value depends on the configuration of the model membrane. For members of the ellipsoid family, the shape determinant can be precisely calculated using the shape formula **(1 - r^2^/2b^2^) **presented in Equation 3. As noted above for a spherical shape this results in a relatively low value of 0.5 due to synclastic membrane elements equally sharing the pressure load. For cylindrical shapes it results in a higher value of 1.0 since uniclastic membrane elements have only a single curvature to bear the pressure load. Thus assigning a spherical configuration to a particular chamber imputes a lower value to its shape determinant while assigning a cylindrical configuration to a chamber imputes a higher value to its shape determinant.

These model shape assignments seem to be reasonable. The accuracy of using these perfect shapes to model imperfect chambers can be assessed using the equations noted above. For example, Equation 3 would predict that an imperfect ampulla with a 10% asymmetry in its diameters would have a computed shape determinant of 0.6 as opposed to 0.5 for a perfect sphere, a fractional departure. In order to assess the effect of using a simple cylinder to model the toroidal semicircular canal, the Equation 1 can be used. The semicircular canal toroids have an external to internal radius ratio of at least 10 in most animals [6]. Such proportions would entail a shape determinant of 0.95 for the outermost synclastic surface and 1.05 for the innermost anticlastic surface, fractional departures from the ideal model value of 1.0.

Thus, values for the shape determinant in the model can be seen to range approximately from 0.5 (sphere) to 1.0 (cylinder). This implies that the overall influence of shape on the geometric stress factor is in the range of two fold (2×) for the model presented.

**Membrane size **is also a linear determinant of hoop stress because the radial dimension of a chamber is simultaneously an inverse measure of surface curvature; the larger the chamber, the greater its radius, the lesser its surface curvature. In theory, size can be unlimited and hoop stress along with it. However it is possible to make some estimate of the size determinant by noting that the vestibular membrane structures are contained in a bony analog, the otic capsule, that acts as a physical limit to the relative sizes that may be attained. Measurements of these otic capsule cavities taken from a wide variety of preserved animal temporal bones indicate that the diameter of the bony channel for the semicircular canal is approximately 1000 μ but can be as large as 2000 μ in the seal, while the diameter of the bony vestibule housing the utricle can be as large as 8 millimeters in the dromedary and 6 millimeters in the whale [6]. Gray makes it a point to indicate that the membranous semicircular canal often fills the bony channel, leaving little in the way of perilymphatic space in most animals. Thus the maximal geometric stress factor disparity between semicircular canal and utricle based on the limiting sizes of these structures could be roughly estimated at 8×.

**Membrane thickness **may be considered the most important passive determinant of hoop stress in that it is the substrate that actually bears the stress load. It has an inverse relationship to stress in that the thinner the membrane the higher the stress level. While the maximum semicircular canal membrane thickness might be estimated at 100 μ (10% of the 1000 μ bone canal noted above], it is the lower limit of membrane thickness that is of importance. In a minimal thickness membrane without any external collagen layer, the stress bearing structure would be the epithelial cell basement membrane which has an approximate thickness of 1 μ. [16] Thus the maximal geometric stress factor disparity due to differences in membrane thickness between chambers could be as much as one hundred fold (100×).

These estimates of the individual contributions of the stress determinants can be used to estimate the potential magnifying effect of geometric stress factor on hoop stress. With a two fold shape effect, an eight fold size effect, and a hundred fold thickness effect, the composite effect of membrane geometry on hoop stress could be as much as sixteen hundred fold. Restated in terms of the model permutations noted above, one could expect a large thin walled cylinder to potentially experience a hoop stress level 1600× that of a small thick walled sphere.

Given this theoretical possibility for large hoop stress disparities to exist in the membranes of the labyrinth due to its complex configuration, it remains to be seen how Nature accommodates this biomechanical vulnerability. Does Nature adjust the configurational determinants of stress to keep hoop stress low and relatively uniform. Does she increase membrane thickness to offset adverse shape or use favorable shape to offset adverse size? Or does Nature tolerate the presence of substantial stress disparities despite the attendant risk of membrane failure. While the final answer to this question must await actual tissue measurements in individual labyrinths, some brief speculations may be in order.

### Speculative Considerations

Nature appears to abhor uniformity in favor of variety when it comes to evolution. The large array of extinct forms in the fossil record, the enormous variety of living species, and the visible differences between the legions of individuals may all be a part of a single evolutionary spectrum. In fact, individual differences in Darwin's view represent the current day manifestation of evolution [17]. And genetic recombination during meiosis [18] virtually guarantees some degree of difference between individuals, whether it be in facial appearance, fingerprint pattern or navigational facility.

Thus some degree of disparity in geometric stress factor might be expected to attend evolutive tendencies in the vestibular membranes. Given the slow pace of evolution, such disparity in geometric stress factor would likely be small in most individuals and of no functional significance. However, substantial disparity in vestibular membrane geometric stress factor would be expected to occur sporadically in individuals [17] and to be attended by significant functional change. The visible changes in the labyrinthine membranes in Meniere's disease [19] may represent evidence of this latter sort. Thus Meniere's disease may provide a window into the process by which configurational membrane changes come about. To the extent that such changes involve the membranes' geometric stress factor, analysis of this factor may provide a tool for investigating the etiology of Meniere's Disease as well as for studying the evolution of the labyrinth itself.

## Conclusion

A model is presented that uses basic geometric shapes to emulate the scheme of the core vestibular membranes. The model is quite flexible in that its component parts can be configured to accommodate any particular species. Analysis of this model shows that the level of static hoop stress induced by trans-mural pressure is a function not only of pressure but of the membrane's structural geometry as well and as such will vary as the dimensions vary. The hoop stress induced per unit of applied pressure can be very large in a thin membrane of very low curvature and, conversely, the hoop stress in a thick highly curved membrane can be quite low, when exposed to the same trans-mural pressure. Indeed hoop stress can vary from point to point within the membrane of a pressurized vessel of irregular shape, depending on the shape, thickness and curvature of the membrane at the point in question. This point is counterintuitive and deserves emphasis. Constant static transmural pressure throughout the labyrinth does NOT imply a constant level of hoop stress in the variously configured containment membranes. The convoluted shape of the membranous labyrinth thus implies a continuum of hoop stress levels throughout its membranes. Such stress disparities may play a role in the development of membrane pathologies as seen in Meniere's Disease. They may also factor in the evolutionary development of other derivative membrane structures such as the saccule, the lagena, and the cochlea found in higher animals.

In sum, this model analysis has identified the approximate shapes of the vestibular chamber membranes, has selected classical geometric shapes of sphere and cylinder to emulate those chambers in a model of the vestibular membranes, has identified a composite dimensional parameter herein termed the geometric stress factor that reflects a membrane's susceptibility to hoop stress, and has shown that this geometric stress factor can be used to analyze hoop stress disparities in vestibular chambers of any species whose real membranes approximate those of the model and whose dimensions are known.

## Competing interests

The author declares that he has no competing interests.

## Authors' contributions

DJP conceived and carried out this analysis and is solely responsible for its content and has read and approved the final manuscript.

## Authors' informations

DJP has a longstanding interest in the architecture of the membranous labyrinth. He completed a fellowship in Otopathology and Electron Microscopy at Harvard Medical School. He has lectured on temporal bone anatomy and disease at the Columbia University Medical Center. He is a physician who specializes in diseases of the ear and holds the position of Associate Clinical Professor of Otolaryngology at Columbia University. He is a member of the New York Otological Society and the International Otopathology Society.
